# Post-vaccine COVID-19 acute myocarditis: case reports and literature review

**DOI:** 10.11604/pamj.2023.44.192.35425

**Published:** 2023-04-20

**Authors:** Abderrahmane Bouchaala, Jaouad Nguadi, Abdelakder Benhlima, Manal Arfaoui, Hamza Elhamzaoui, Mustapha Alilou

**Affiliations:** 1 Emergency Department, Ibn Sina Hospital, Faculty of Medicine and Pharmacy in Rabat, Rabat, Morocco

**Keywords:** Acute myocarditis, COVID-19, COVID-vaccines, case report

## Abstract

COVID-19 vaccines have reduced both lethality and hospitalization rates of the novel coronavirus disease. Nevertheless, multiple side effects have been reported in the literature, most often are harmless. We report two cases of acute myocarditis, hospitalized in the emergency department for chest pain occurring after the second dose of mRNA vaccine AstraZeneca. The SARS-Cov-2 infection was ruled out in both patients with a negative PCR obtained by nasal swabs and normal thoracic CT scans. Both patients had high levels of high-sensitive cardiac troponin I. Acute coronary syndromes were excluded with cardiac catheterization. Cardiac Magnetic resonance imaging (MRI) showed signs in favor of acute myocarditis. The evolution was favorable for both patients after being put on anti-inflammatory treatment. The universality and accumulation of reports concerning acute myocarditis following COVID vaccination, in the absence of any other diagnostic element that could explain the myocardial injury, establish a strong causal link, although the etiopathogenesis of such injury remains poorly elucidated.

## Introduction

As with many types of vaccines, such as smallpox and seasonal influenza vaccines [1,2], there have been several reports of post-vaccination myocarditis with different types of COVID-19 vaccines [3,4]. We present the case of two young patients, aged 26 and 46 years, who developed acute myocarditis within two weeks after the second dose of mRNA anti-COVID-19 vaccines.

## Patient and observation

**First patient information:** patient A.S., 26 years old, with no cardiovascular risk factors or significant pathologic history, admitted to the emergency department for angina pain occurring after 14 days of the second dose of the Covid-19 vaccine type Vaxzevria (Oxford/AstraZeneca vaccine). The patient had no contact with a COVID-19 positive case, no recent influenza-like illness and a negative SARS-Cov-2 PCR on admission.

**Clinical findings:** at admission, physical examination found the patient to be in good general health, apyretic with a heart rate of 75 beats per minute, eupneic with a respiratory rate of 16 cycles per minute and a blood pressure of 131/74 mmHg. There was no evidence of heart failure. Peripheral pulses were present and symmetrical on palpation. Cardiac, pulmonary and arterial auscultation were unremarkable. The examination of the other systems, notably the neurological, pleuropulmonary and digestive systems, was unremarkable.

**Figure 1:**
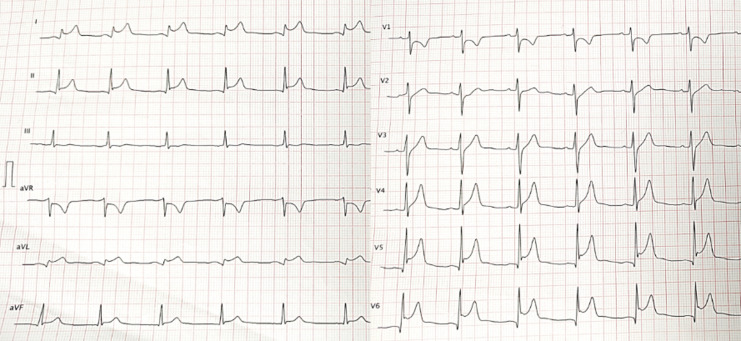
electrocardiogram showing inferolateral ST-segment elevation

**Figure 2:**
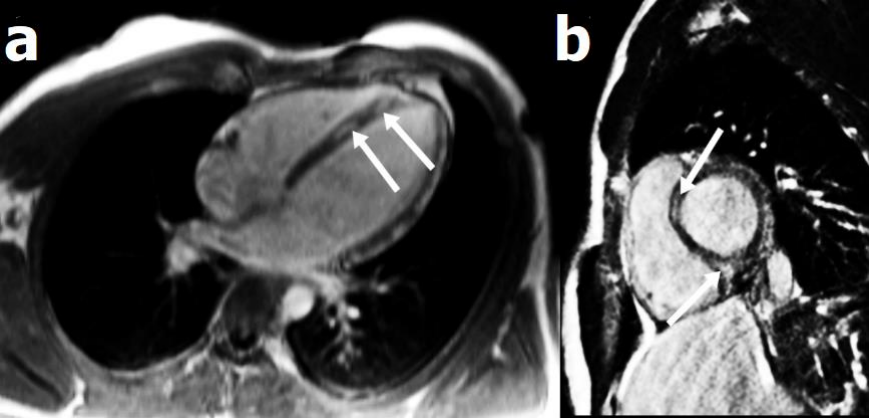
four-cavity (a) and short-axis (b) cardiac MRI showing late gadolinium enhancement in septal wall, suggestive of myocarditis

**Diagnostic assessment:** the initial ECG showed an inferolateral ST-segment elevation (Figure 1). The ultra-sensitive troponin I level was 6700 ng/L (normal value NV < 40 ng/L). Coronary angiography did not show any coronary network lesion. Markers of inflammation were elevated, with CRP at 230 (VN <6 mg/L) and leukocytes of 17,700/mm^3^. The viral respiratory panel, including SARS-Cov-2 PCR, was negative. Echocardiography did not show any particularity. Cardiac MRI showed increased signal density on T2 weighted sequences with late enhancement at the septo-apical level Figure 2. The diagnosis of acute myocarditis was retained. Other laboratory results indicated a hemoglobin of 14.8 g/dL, platelets of 330,000/mm^3^, blood sugar of 107 mg/dL, sodium of 141 mmol/L, potassium of 4.2 mmol/L and normal urinalysis.

**Therapeutic intervention:** the evolution under treatment with non-steroidal anti-inflammatory drugs (NSAIDs) was marked by clinical and biological improvement. The troponin level decreased by half on day 3 and was normalized by day 5.

**Follow-up and outcomes:** to date, the patient remains asymptomatic, in good clinical condition, with no reported complications or recurrence.

**Informed consent:** written informed consent, dated and signed was obtained from the patient.

**Second patient information:** patient S.H, 46 years old, with no notable pathological history, was vaccinated 15 > days before her admission with the anti-COVID-19 vaccine Vaxzevria (Oxford/AstraZeneca). She was admitted to the emergency department for chest pain associated with palpitations and asthenia. The patient had no history of contact with Covid-19 or recent influenza-like illness.

**Clinical findings:** physical examination revealed a tachycardia at 160 beats/min that was hemodynamically well tolerated with no signs of peripheral hypoperfusion or heart failure. Patient was eupneic with a respiratory rate of 20 cycles per minute. The examination of the other systems was unremarkable.

**Figure 3:**
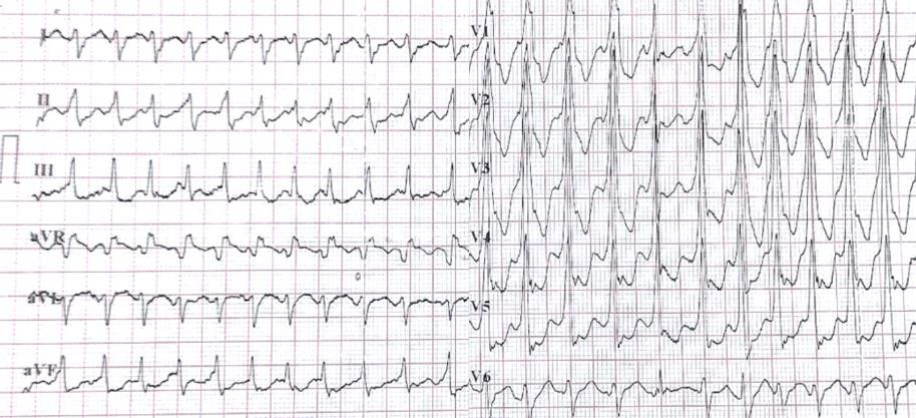
electrocardiogram showing ventricular tachycardia

**Diagnostic assessment:** the ECG showed ventricular tachycardia (Figure 3). Biologically, the ultra-sensitive Troponin I level was 1.970 ng/L (normal value NV < 40 ng/L), CRP was 24.3mg/L, kalemia was 4.2 mmol/L, and TSH was 1.06 mIU/L. The respiratory panel for other viral infections responsible for the myocarditis was negative, as was the SARS-Cov-2 PCR obtained by nasal swab. Echocardiography and coronary angiography were unremarkable. Cardiac MRI showed T1 weighted early and late subepicardial enhancement at the apex suggestive of myocarditis.

**Therapeutic intervention**: the patient was initially put on Lidocaine with rapid return to a regular sinus rhythm. The patient was put on analgesics and corticosteroid therapy based on methylprednisolone at 2mg/Kg for 7 days, then a progressive reduction over three weeks. The clinical and biological evolution was favorable.

**Follow-up and outcomes:** to date, the patient has not reported any symptomatic recurrence, in good clinical condition, with no reported complications.

**Informed consent:** written informed consent, dated and signed was obtained from the patients.

## Discussion

We report two cases of acute symptomatic myocarditis occurring after the second dose of Vaxzevria vaccine (Oxford/AstraZeneca vaccine), in the absence of any clinical, biological or radiological evidence of concomitant SARS-CoV-2 infection and a negative etiological investigation. The occurrence of post-vaccine acute myocarditis or perimyocarditis is a rare event but has been reported sporadically in the literature. Prior to the COVID-19 era, acute myocarditis constituted approximately 0.1% of adverse events reported to the Vaccine Adverse Event Reporting System (VAERS) in the United States between 1990 and 2018 [5]. Since the introduction of COVID-19 vaccination, several cases of acute myocarditis occurring after vaccination have been reported [3,4,6]. For example, the Centers for Disease Control and Prevention CDC Advisory Committee on Immunization Practices reported a rate of 3.5 cases of myocarditis per million second doses of the various mRNA vaccines administered [7]. Similarly, the US Army reported 23 cases of myocarditis among 2.8 million doses of mRNA vaccines administered [8]. Several hypotheses tried to explain the etiopathogenic mechanisms of post-vaccination myocarditis. One of them is the involvement of anti-idiotype antibodies in a cross-reaction with myocyte cells, leading to apoptosis and inflammation of the myocardium. RNA vaccines are also suspected of triggering an innate inflammatory response responsible for myocardial damage [9]. The intramuscular injection method, without prior aspiration, with the possibility of air bubble injection has been widely discussed.

The time between vaccination with COVID-19 and the onset of symptoms related to myocarditis varies considerably between individuals. The two cases we report presented clinical signs 2 weeks after the second dose of Vaxzevria vaccine (Oxford/AstraZeneca). In the literature, almost all cases of myocarditis have been reported within one week of vaccination, with an average duration of three days [10]. Other reports have highlighted a longer delay of up to 40 days, suggesting the involvement of the composition of the different vaccines and the individual immune response [11]. The diagnosis of acute myocarditis remains a challenge due to the non-specificity of the symptoms. Given the diversity of clinical presentations, ranging from simple chest pain to unexpected cardiogenic shock, and the specific increase in cardiac biomarkers, cardiac MRI has been shown to be essential for diagnostic confirmation and topographical characterization, although endomyocardial biopsy remains the golden standard for histological certainty [12]. The therapeutic management of patients with acute post-vaccinal myocarditis and perimyocarditis is based on the use of NSAIDs, glucocorticoids and colchicine in the first line of treatment for several authors. Other reports suggest the use of immunoglobulins, beta-blockers, and converting enzyme inhibitors in systolic ventricular dysfunction. Although studies on the treatment of these patients are lacking, these therapies should be considered, especially in patients with significant symptomatology.

## Conclusion

Post-vaccination induced myocarditis has been reported sporadically in the literature long before the era of COVID-19. The occurrence of this pandemic has made large-scale vaccination a global health emergency. While several reports have highlighted the onset of myocarditis and/or pericarditis within days of the second dose, the low incidence of this side effect and the innocuousness of the symptoms made COVID-19 vaccines overall safe, taking into account that this risk has to be weighed against the much greater risk of death, respiratory, cardiovascular, and systemic complications from SARS-CoV-2 itself.
